# The Epidemiological features of lymphoid malignancies in Benin City, Nigeria: a 15 years study

**Published:** 2012-01-20

**Authors:** Caroline Edijana Omoti, Alexander Ikenna Nwannadi, Jude Chike Obieche, Adesuwa Noma Olu-Eddo

**Affiliations:** 1 University of Benin Teaching Hospital P.M.B. 1111, Benin City, Nigeria

**Keywords:** Epidemiology, lymphoma, leukemia, myeloma, morphology, Nigeria

## Abstract

**Introduction:**

Lymphoid malignancies compose a wide spectrum of different morphologic and clinical syndromes known to vary widely throughout the world. The purpose of this study is to determine the prevalence and time trends of lymphoid malignancies.

**Methods:**

A 15 (May 1st 1996-April 30th 2010) years study of all patients who had lymph node biopsy at the Department of Haematology and Pathology, University of Benin Teaching Hospital, Benin City, Nigeria.

**Results:**

The 391 patients had a male preponderance (M:F; 1.6:1). An increase in the lymphoid malignant cases was noted from 95 cases in the first 5-year interval (1996–2000) to 179 cases in the last 5-year interval (2006–2010) giving an average increase of 84.0%. Non-Hodgkins lymphoma (61.1%) and chronic lymphocytic leukaemia (18.2%) were the most frequent followed by Hodgkin's lymphoma and myeloma with equal proportions of 9.0% each. A positive correlation with a significant linear trends was obtained (r=0.1949, p<0.0001). Geographic areas at risk were found mainly in patients residing in Delta State (67.0%) which is a major oil producing state and Edo State (30.4%) where the hospital is located, both in the Niger Delta Region of Nigeria.

**Conclusion:**

Future research into environmental agents and genetic makeup/HLA typing of patients can be carried out.

## Introduction

Lymphoid malignancies compose a wide spectrum of different morphologic and clinical syndromes known to vary widely throughout the world. Though the incidence of these malignant tumors has increased steadily worldwide the cause of most lymphoid neoplasms remain unknown [1]. Previous reports have even indicated that patients with haematologic malignancies (e.g chronic lymphocytic leukaemia, non-Hodgkin's lymphoma) may be at increased risk of second neoplasms [2]. Investigation of trends in incidence rates of lymphoid malignancies is essential for the objective assessment of health consequences because of increasing industrialization and urban westernization including lifestyle changes. The relative frequency of the occurrence of different histologic and immunophenotypic subtypes varies according to race and geography and this may be ascribed to genetic and environmental aetiologic factors [3]. The analysis of lymphoid malignancies in a single institution at different periods of time can determine the changing status of the disease in this region. Hence, comparison of incidence and patterns by disease subtype may provide critical clues for future aetiologic investigation. We therefore aim in this study to determine the prevalence and time trends of lymphoid malignancies in Benin City, Nigeria.

## Methods

The case files of all haematologic admission were extracted by multiple information sources from the medical and histological records of University of Benin Teaching Hospital (UBTH), Benin City and recognized pathology laboratories in Nigeria from May 1st 1996- April 30th 2010. The detailed study design is that UBTH is a major referral centre serving the South-South geopolitical zone of Nigeria. Patients from the catchment region of the country have access to the hospital which is a government based hospital and one of the best known Medical Schools in Nigeria. Those with diagnosis of lymphoid malignancies were identified. The temporal trend of prevalence rates and their relation with age, gender and histological types of lymphoid neoplasms were studied. The pretherapy, clinical, socioeconomic status and demographic features were analysed. The total number of lymphoid malignant cases for each year was specifically noted while the total number of patients with lymphoid malignancies in the same time interval was calculated.

Tissue samples were studied by histological examination of surgical biopsy from an accessible nodal site by the Histopathology department. Diagnosis was established based on clinical information and cytomorphological diagnostic criteria as recommended by the National Cancer Institute (NCI) [4] including well stained peripheral blood film by a Romanowsky stain, bone marrow aspiration cytology and trephine biopsy. For prolymphocytic leukaemia (PLL) diagnosis was according to Melo et al [5]. The diagnostic criteria applied for myeloma is in keeping with the Myeloma Task Force Criteria [6] while chronic lymphocytic leukaemia (CLL) is according to Rai [7] and Binet staging [8]. The acute lymphoblastic leukaemia (ALL) subtype was based on the morphological criteria of the French- American- British (FAB) co-operative group [9]. Diagnosis of non-Hodgkin's lymphoma was based on the working formulation. Other laboratory studies include complete blood count, Coombs test, immunologic studies which determine monoclonality, serum protein electrophoresis, blood cultures, serum electrolyte and urea, ultrasonography.

### Data analysis

Data was analyzed using Instat GraphPadTM version 2.05a statistical software. The statistical methods applied include frequency counts and cross tabulations using Yates correction whenever necessary and one-way analysis of variance (ANOVA) for significant association. The results are presented as mean±SD, median and range. P-value of

## Results

The total number of Haemato-oncology admissions between 1996 and June 2010 was 1,081 (721 males and 360 females) comprising at five year intervals of 299 (May 1996–2000), 360(2001–2005) and 422 (2006-June 2010). Out of these, the total number of patients with lymphoid malignancies admitted and those seen at the outpatients clinic was 391 comprising 95 (24.3%), 117(29.9%) and 179 (45.8%) respectively which reveals a progressive time increase in lymphoid malignant cases over the 5-year interval periods. The total bed capacity of the hospital is 680 while the catchment population of the hospital is a total population of about 12 million.

**Figure 1:**
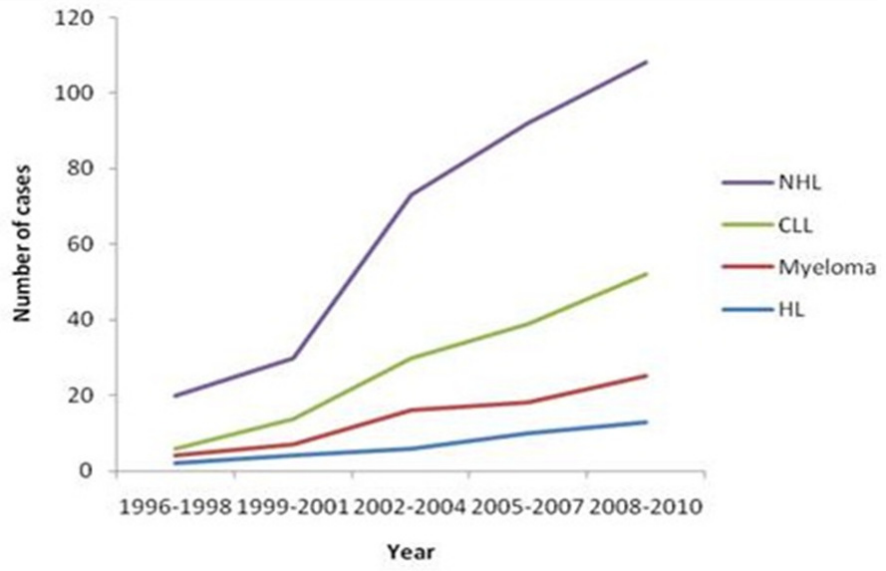
Distribution trends of the major four lymphoid malignant cases seen during the study period

An increase in the lymphoid malignant cases was noted from 95 cases in the first 5-year interval (1996-2000) to 179 cases in the last 5-year interval (2006-2010) giving an average increase of 84.0%. Overall an increase in the number of cases was observed. Figure 1 depicts the progressive time trends in prevalence of the four major lymphoid malignancies encountered in this study at three-year intervals. The mean overall number of cases for the 3-year interval was 17.5±4.2, 18.9±5.1 and 23.7±6.4 with a median of 17.5, 19.5 and 24.5 respectively. The relationship was such that there was an increasing trend and that most affected subjects were aged 46–59 years or younger in the latter years (2006–2010) compared to a decline in the older patients (>60 years) for the period studied.

### Prevalence according to age and gender

The 391 patients comprised 238 males (60.9%) and 153 females (39.1%) with a male to female ratio of 1.6:1. The overall median age was 41.0 years (range; 1–85 years) with a mean age of 40.0±21 years (SE±1.1). The relationship between the age and sex distribution of patients with lymphoid malignancies for the various 5-year interval is shown in Table 1. The most frequent age group was 46-59 years with 36.6% less than 45 years and 19.7% within the paediatric age group (1–17years). Detailed overview of the histological diagnosis and the mean age statistical analysis of lymphoid malignant subtypes are shown in Table 2. The mean age of non Hodgkin's lymphoma (NHL) patients was 52.5±22.1 years for males and 49.5±22.3 years for females while for Hodgkin's lymphoma (HL), the mean ages were 35.4±20.0 years in males and 35.8±12.5years in females. For chronic lymphocytic leukaemia (CLL) the mean age was 59.1±17.3 years and 56.0±7.2 years for males and females respectively. Burkitt's lymphoma (BL) which was frequent in the paediatric age group had a mean age of 6.7±3.8 years for males and 8.9±2.9 years for females.

**Table 1:** The relationship between the age and sex distribution of patients with lymphoid malignancies for the various year interval

	1996 – 2000		2001 – 2005		2006 – 2010		Total
	
Age (years)	M	F	M	F	M	F	
1–17	8	16	19	5	18	11	77
18–31	12	4	16	5	25	10	72
32–45	10	7	11	10	21	12	71
46–59	16	9	11	17	29	17	99
60–73	7	6	13	8	14	7	55
74–80	1	3	1	0	4	4	13
>80	1	0	0	1	1	1	4
**Total**	**55**	**45**	**71**	**46**	**112**	**62**	**391**

**Table 2:** Detailed histological diagnosis, Incidence, sex ratios and statistical analysis of lymphoid malignant subtypes

Histological diagnosis	n	%	Male	Female	Mean	Std dev	Age range
**Lymphomas**							
NHL	239	61.1	171	68	52.5	22.1	1-83
HL	35	9.0	21	14	32.9	17.7	9-66
**Leukaemias**							
CLL	71	18.2	32	39	55.9	13.3	24-84
ALL	34	8.7	19	15	21.2	11.7	4-71
PLL	22	5.6	11	11	61.8	17.0	26-79
HCL	12	3.1	7	5	51.1	7.8	39-60
**Myeloma**	35	9.0	18	17	58.0	8.5	42-70

NHL- Non- Hodgkin's lymphoma; HL- Hodgkin's lymphoma; ALL-Acute lymphoblastic leukaema; CLL- Chronic lymphocytic leukaemia; HCL- Hairy cell leukaemia; PLL- Prolymphocytic leukeamia

### Relative frequency of the lymphoid malignancies

Analysis of the lymphoid neoplasms revealed that NHL (61.1%) and CLL (18.2%) were the most frequent followed by HL and myeloma with equal proportion of 9.0% each. The other lymphoid malignancies include acute lymphoblastic leukaemia 34 (8.7%), prolymphocytic leukaemia 22 (5.6%) while the least frequent lymphoid malignancy was hairy cell leukeamia (HCL) in 12 patients (3.1%). A male preponderance (M:F) of 2.5:1, 1.5:1,, 1.3:1 and 1.4:1, was found for NHL, HL, ALL and HCL respectively while a slight female preponderance (M:F) of 1:1:2 and 1:1.1 was found for CLL and myeloma respectively as shown in Table 2. In both men and women, the more frequent lymphoid malignancies were NHL and CLL while the 3rd most frequent malignant disorder in males was BL and in females was myeloma.

### Demographic characteristics

Table 3 shows the patients socioeconomic and demographic profile at presentation. The majority of the patients were married (68.8%), some were single (27.6%) while 2.0% and 1.5% were widowed and divorced respectively. A total of 354 patients (88.0%) were Christians, 20 (5.1%) were Muslims and 17(4.3%) atheists. Geographic areas at risk were found mainly in patients residing in Delta State (66.5%) which is one of the major oil producing state and Edo State (30.4%) where the hospital is located, both in the Niger Delta Region of Nigeria. The remaining 3.1% were other neighboring states which are still located within the oil producing Niger Delta region of Nigeria. This was further confirmed by the Urhobo/isoko ethnic groups in Delta state and the Bini in Edo state recording the highest prevalence of lymphoid malignancies. Close observation revealed that the more educated patients (59.6%) presented with lymphoid malignancies. On the whole, 66 patients (16.9%) had no formal education, 96 patients (24.6%) had primary education and 119 patients (30.4%) had secondary education while 110 patients (28.1%) had a tertiary form of education. A marginally higher risk of lymphoid neoplasm was observed for the lower socioeconomic categories (51.9%). Of the 391 patients, 82 (21.0%) were civil servant, 81 (20.7%) traders, 61 (15.6%) each were farmers and artisans while the largest socioeconomic class 106 (27.1%) were unemployed.

**Table 3:** The sociodemographic characteristic of 391 patients with lymphoid malignancies during the study period

Variables	Frequency	(%)
**Sex**		
Male	238	60.9
Female	153	39.1
		
**Marital status**		
Single	108	27.6
Married	269	68.8
Widowed	8	2.0
Divorced	6	1.5
		
**Geographical Abode**		
Edo	119	30.4
Delta	260	66.5
Others	12	3.1
		
**Ethnic Group**		
Bini	150	38.4
Urhobo/Isoko	170	43.5
Others	71	18.2
		
**Educational status**		
Illiterate	66	16.9
Primary	96	24.6
Secondary	119	30.4
Tertiary	110	28.1
		
**Occupational Status**		
Civil Service	82	21.0
Trading	81	20.7
Artisan	61	15.6
Farming	61	15.6
Unemployment	106	27.1

## Discussion

Despite proliferation of epidemiological studies during the past two decades, aetiologies of lymphoid malignancies remain poorly understood and characterization of descriptive patterns has been limited [10]. The increasing incidence rate of lymphoid malignancies may be partially explained by improved diagnostic skills and health facilities.

Non-Hodgkin's lymphoma (61.1%) and CLL (18.2%) were the most common lymphoid malignancies in this study. The high prevalence of lymphoma observed correspond to the incidence rates observed in Europe, Africa and throughout the world [11,12]. A similar study carried out in equatorial belt of Africa showed that lymphomas were among the top-ten tumors in the geographical region where pathogens and environmental factors have been detected suggesting that they may contribute to lymphomagenesis. [13]. Bhurgri et al [14] reported an increasing incidence and time trends in a population-based study carried out in Karachi. However, the incidence of lymphomas as reported from the Kuwait cancer control center registry was said to have declined [15]. This difference in time trends may have a genetic basis and differing exposure to environmental agents. Current evidence suggests that factors and conditions that precipitate either chronic antigenic stimulation or immunosuppression may provide a preferential milieu for development of NHL [16].

The increase in total number of lymphoid malignancies from 90 cases to 164 cases in the time interval studied is similar to the pattern of an increasing number and incidence for the various lymphoid subtypes in another study [17]. In the largest series of lymphoma cases ever published from a single institution in Thailand over a 15 year period, there was an increase in lymphoma cases from 73 cases per year in the first series to 189 cases per year in the second series (an increase of 158.9%) [17].The increasing trends of malignancies were attributed to a number of factors: First, there is a better histologic evaluation in conjunction with immunohistochemical studies which play an important role in diagnosis. Secondly, improved standard of health care system which provide easier patient access to the hospital. The increasing prevalence may also be attributed to the increasing incidence of HIV in sub-Saharan African and the use of immunosuppressive therapy. The low prevalence of cases seen beyond the age of 80 years is probably due to the peculiar demographic distribution of the population after that age.

Although the sex of an individual confers one of the greatest known risk for contracting lymphoma and leukaemias, very little attention is paid to these risks [18]. It has been reported that the male excess in lymphoid cancer is most marked in the youngest age group in NHL and HL while ALL shows equal sex ratio in childhood peak [18] or slightly higher male ratio as reported by Rodriquez-Abreu et al [19]. The latter finding was observed in this study. The predominance of male in the lymphoid cases was seen in this study except for CLL where female dominance and elderly occurrence was further reinforced with a median age of 59 years for males and 56 years for females. The female preponderance recorded in CLL has been reported in an earlier study in the same institution [20] and Western region of Nigeria [21]. This difference may probably be due to racial or genetic factors. Also notable was that the median age of patients at the time of presentation was younger than in Korea and US (45years vs 52years vs 65years) respectively [22]. This might be attributed to the lower frequency of follicular lymphoma that tends to occur in older patients which was higher in the other study [22]. The male predominance could be attributed to the fact that males are more exposed to occupational agent that predispose to development of malignancies than females. There is also the possibility that males may have more unstable genetic composition that does not withstand genetic injury as in females. It may also be that males have a weaker DNA repair apparatus than the females. These are areas that will require future research.

In the developed world, classification of lymphomas is based on morphologic and immunophenotypic features. Unfortunately, immunohistochemistry is not routinely done in our centre, therefore constituting a major limitation in the classification of these malignancies. The prevalence of follicular lymphoma in our series was low (3.3%) when compared with that encountered in the US [23] and Thailand [17] but was similar to that reported by Intragumorchai et al [24] in another study in Thailand (3.8%). Burkitt's lymphoma is a common malignancy in tropical Africa and predisposing factors include malaria and infection with the Epstein-Barr virus. Recent studies suggest that the prevalence of this neoplasm is declining in this environment. In Ibadan, another community in the Western region of Nigeria it seems that what appear to be minor changes might actually be a real decline in the incidence of BL and that it might be partly ascribed to improved living conditions and greater control of malaria. Myeloma made up to 9.0% of the lymphoid malignancies and was the 3rd most frequent malignant disorder in women in this study which is similar to the study carried out in France [25]. This value is higher than the value obtained in other studies in Africa [21] but however lower than what was obtained in West Indies [26]. Hairy cell leukemia with the least incidence of 3.1% had a male preponderance and is known to have a poor prognosis. In a 10 year survey of HCL in the same institution a total of 0.6 cases/year was recorded [27] as compared to 0.8 cases/year in this 12 year study with less favorable prognosis.

Socioeconomic constraints have been known to adversely affect the management of lymphoid malignancies in Nigeria [28,29]. Though there is a selection bias as it does not represent a true community based report, majority of the patients were found to reside in Delta State, a major oil producing state where there are petrochemical industries and gas flare sites. Even the other patients were found to reside in other parts of the Niger Delta region of the country. The related environmental pollution from its refinery and petrochemical industries along with yet to be confirmed toxic waste deposition and radioactive waste products may have contributed to substantial number of lymphoid malignancies from this region. However, other aetiologic factors including genetic and environmental influences have been identified in the past as causes of lymphoma especially [30,31]. The increase time trend apart from environmental influences and hazards could also be attributed to other factors: poverty, decreased immunosurveillance from problems of under nutrition and malnutrition. It is also possible that some of the increase in the number of cases of lymphoid malignancies may be due in part to the increase in population in Nigeria.

The increasing tendency for people to work outside their home community'one of the most striking of modern demographic changes-has relevance to a recent aetiological hypothesis about childhood leukaemia: that a community's immune response to an underlying infection can be disturbed by increases in new social contacts [32]. Unemployment which accounted for the highest occupational status include students, applicant and pensioners. The various agents in the environment to which subsistence farmers and artisan workers are exposed to has been implicated in aetiology of lymphoid malignancies [33]. In Italy, researchers have also demonstrated that farmers and industrial workers have a significant risk for haematological malignancies [33].

## Conclusion

In conclusion, our study shows an increasing time trend in lymphoid malignancies in Nigeria with NHL and CLL being the most frequent. The lymphoid malignancy is a disease of the young to middle age group occurring mainly in geographic areas of oil producing states. The significant increase in the number of cases and male excess in lymphoid malignancies requires further study regarding lymphomagenesis including research into environmental agents that may be implicated.
